# Study on the inhibition activity and mechanism of Tanreqing against *Klebsiella pneumoniae* biofilm formation *in vitro* and *in vivo*

**DOI:** 10.3389/fcimb.2024.1368450

**Published:** 2024-04-04

**Authors:** Wenxia Zhang, Min He, Nana Kong, Yuxiao Niu, Anhong Li, Yuzhong Yan

**Affiliations:** ^1^ Department of Laboratory Medicine, Zhoupu Hospital, Shanghai University of Medicine & Health Sciences, Shanghai, China; ^2^ Clinical Research Center, Shuguang Hospital, Shanghai University of Traditional Chinese Medicine, Shanghai, China; ^3^ Department of Clinical Laboratory, Longhua Hospital Affiliated to Shanghai University of Traditional Chinese Medicine, Shanghai, China; ^4^ Xinxiang Medical University, Xinxiang, Henan, China; ^5^ Shanghai University of Traditional Chinese Medicine, Shanghai, China

**Keywords:** Tanreqing, *Klebsiella pneumoniae*, biofilm, quorum sensing, lung infection

## Abstract

**Objective:**

To evaluate the antibacterial effect of Tanreqing (TRQ) against *K. pneumoniae* and its inhibition activity on bacterial biofilm formation *in vitro* and *in vivo*, and to explore the mechanism of the inhibitory effects of TRQ on *K. pneumoniae* biofilm formation.

**Methods:**

An *in vitro* biofilm model of *K. pneumoniae* was established, and the impact of TRQ on biofilm formation was evaluated using crystal violet staining and scanning electron microscopy (SEM). Furthermore, the clearance effect of TRQ against *K. pneumoniae* in the biofilm was assessed using the viable plate counting method; q-RT PCR *w*as used to evaluate the inhibitory effect of different concentrations of TRQ on the expression of biofilm-related genes in *Klebsiella pneumoniae*; The activity of quorum sensing signal molecule AI-2 was detected by *Vibrio harveyi* bioluminescence assay; Meanwhile, a guinea pig lung infection model of *Klebsiella pneumoniae* was constructed, and after treated with drugs, pathological analysis of lung tissue and determination of bacterial load in lung tissue were performed. The treatment groups included TRQ group, imipenem(IPM) group, TRQ+IPM group, and sterile saline group as the control.

**Results:**

The formation of *K. pneumoniae* biofilm was significantly inhibited by TRQ *in vitro* experiments. Furthermore, when combined with IPM, the clearance of *K. pneumoniae* in the biofilm was notably increased compared to the TRQ group and IPM group alone. q-RT PCR analysis revealed that TRQ down-regulated the expression of genes related to biofilm formation in *K. pneumoniae*, specifically *luxS*, *wbbm*, *wzm*, and *lsrK*, and also inhibited the activity of AI-2 molecules in the bacterium. *In vivo* experiments demonstrated that TRQ effectively treated guinea pig lung infections, resulting in reduced lung inflammation. Additionally, when combined with IPM, there was a significant reduction in the bacterial load in lung tissue.

**Conclusion:**

TRQ as a potential therapeutic agent plays a great role in the treatment of *K. pneumoniae* infections, particularly in combination with conventional antibiotics. And TRQ can enhanced the clearance effect on the bacterium by inhibiting the *K. pneumoniae* biofilm formation, which provided experimental evidence in support of clinical treatment of TRQ against *K. pneumoniae* infections.

## Introduction

*K. pneumoniae* is a common nosocomial pathogen in hospitals. According to the monitoring data from the Chinese CHINET Bacterial Resistance Surveillance Network, the isolation rate of *K. pneumoniae* has been increasing annually. The resistance rates to the carbapenem antibiotics meropenem and imipenem have risen from 2.9% and 3.0% in 2005 to 27.10% and 26.2% in 2019, respectively (Liang et al., 2021). Among the top ten global health threats announced by the WHO in 2019, diseases closely related to infections occupy the top six positions with an absolute advantage, and the issue of antimicrobial resistance is ranked second. Evidently, antimicrobial resistance and infections have garnered global attention. Therefore, it is urgent to find effective antibiotics that are less prone to inducing resistance to meet the increasing demand for treating infections caused by resistant bacteria.

Studies have shown that almost all *K. pneumoniae* can form biofilms, especially those with high resistance rates (Vuotto et al., 2017; Rahdar et al., 2019). Biofilm formation is one of the most important virulence characteristics of *K. pneumoniae*. Bacteria in biofilms can activate virulence factors, express resistance genes, and mediate plasmid DNA transfer through quorum sensing systems. Biofilms also possess the ability to resist phagocytosis and evade the host’s self-defense mechanisms, thereby promoting the occurrence and development of bacterial infectivity and resistance (Vestby et al., 2020). This makes it more difficult to eradicate infections caused by such biofilm-forming resistant bacteria.

TRQ is a widely used classical compound preparation in China, composed of five Chinese herbs: Scutellaria baicalensis, bear bile powder, horn of Capra hircus, Lonicera japonica, and Forsythia suspense according to the principles of compatibility in traditional Chinese medicine. It has broad-spectrum antibacterial, anti-inflammatory, antiviral, antipyretic, expectorant, and immunomodulatory effects (Liu et al., 2016; Yang et al., 2018). *In vitro* experiments have confirmed that TRQ has a good inhibitory effect on multidrug-resistant bacteria such as *K. pneumoniae*, *Staphylococcus aureus*, and *Acinetobacter baumannii* (Pan et al., 2016; Yang et al., 2018). Numerous clinical trials have also demonstrated that TRQ has a very good therapeutic effect on lung infections caused by resistant bacteria (Yang et al., 2022), especially when used in combination with western medicine, which can significantly improve efficacy (Wang et al., 2020b; Wang et al., 2021; Zhou et al., 2021; Li et al., 2022). Its mechanism of action is complex and diverse. Some studies suggest that TRQ targets cell division to inhibit cell growth while exerting an anti-infective effect by down-regulating the expression of virulence factors (Yang et al., 2022). Other studies have reported that TRQ combined with vancomycin or linezolid has a synergistic effect against MRSA biofilm formation (Yang et al., 2018). However, there are currently no reports on the effects of TRQ alone or in combination with other antibiotics on *K. pneumoniae* biofilm formation.

The objective of this study was to construct an *in vitro* biofilm model of *K. pneumoniae* to assess the impact of TRQ alone or in combination with IPM on biofilm formation by *K. pneumoniae* previously isolated by our research group with biofilm-forming capabilities. Through the analysis of biofilm-related gene expression and quorum sensing molecule activity, we aimed to investigate the mechanism of TRQ against *Klebsiella pneumoniae* biofilm formation. Additionally, a guinea pig pulmonary infection model was established to further validate the *in vivo* anti-infective effect of TRQ. This provided theoretical evidence and reference for the role of TRQ in anti-biofilm formation of *K. pneumoniae* and its treatment of related infectious diseases.

## Materials and methods

### Bacterial strains and reagents

Clinical specimens of biofilm-producing *K. pneumoniae* isolated from hospitalized patients during experiments conducted by our research group from January to December 2020 were selected. The details of the strains were shown in **Table 1**. These strains were preserved in an ultra-low temperature freezer using 20% glycerol broth. TRQ was purchased from Shanghai Kaibao Pharmaceutical Co., Ltd., with the National Medical Product Approval Number Z20030054.

**Table 1:** The details of the strains.

Isolates	Organism	specimen	β-lactamase	MIC	
				IPM(mg/L)	TRQ(ul/ml)
K1	*K. pneumoniae*	sputum	KPC-2	64	500
K2	*K. pneumoniae*	sputum	KPC-2	32	500
K3	*K. pneumoniae*	sputum	KPC-2	256	500
K4	*K. pneumoniae*	urine	KPC-2	256	250
K5	*K. pneumoniae*	bile	NDM	16	250
K6	*K. pneumoniae*	urine	KPC-2	128	125
K7	*K. pneumoniae*	sputum	KPC-2	64	125
K8	*K. pneumoniae*	urine	KPC-2	64	250
K9	*K. pneumoniae*	blood	KPC-2	128	500
K10	*K. pneumoniae*	BALF	KPC-2	64	125
K11	*K. pneumoniae*	urine	KPC-2	64	500
K12	*K. pneumoniae*	blood	KPC-2	64	250
K13	*K. pneumoniae*	sputum	NDM	32	125
K14	*K. pneumoniae*	sputum	KPC-2	128	125
K15	*K. pneumoniae*	bile	KPC-2	256	62.5
K16	*K. pneumoniae*	Anal swab	KPC-2	128	500
K17	*K. pneumoniae*	sputum	KPC-2	128	250
K18	*K. pneumoniae*	sputum	KPC-2	64	62.5
K19	*K. pneumoniae*	blood	KPC-2	128	125
K20	*K. pneumoniae*	urine	KPC-2	64	500
K21	*K. pneumoniae*	BALF	KPC-2	64	250
K22	*K. pneumoniae*	Anal swab	KPC-2	32	250
K23	*K. pneumoniae*	sputum	NDM	32	125
K24	*K. pneumoniae*	sputum	KPC-2	256	500
K25	*K. pneumoniae*	sputum	KPC-2	128	500
K26	*K. pneumoniae*	secretion	KPC-2	64	125
K27	*K. pneumoniae*	sputum	KPC-2	64	500
K28	*K. pneumoniae*	sputum	KPC-2	64	500
K29	*K. pneumoniae*	Anal swab	KPC-2	256	125
K30	*K. pneumoniae*	sputum	KPC-2	32	250
K31	*K. pneumoniae*	BALF	KPC-2	128	125
K32	*K. pneumoniae*	pus	KPC-2	128	62.5
K33	*K. pneumoniae*	sputum	KPC-2	64	500
K34	*K. pneumoniae*	sputum	KPC-2	32	250
K35	*K. pneumoniae*	sputum	NDM	16	250
K36	*K. pneumoniae*	BALF	NDM	16	250
K37	*K. pneumoniae*	sputum	KPC-2	32	250
K38	*K. pneumoniae*	Anal swab	KPC-2	256	250
K39	*K. pneumoniae*	cerebrospinal fluid	KPC-2	128	500
K40	*K. pneumoniae*	sputum	KPC-2	64	125
K41	*K. pneumoniae*	sputum	KPC-2	64	62.5
K42	*K. pneumoniae*	sputum	KPC-2	256	250
K43	*K. pneumoniae*	sputum	KPC-2	256	250
K44	*K. pneumoniae*	ascites	KPC-2	64	500

### Bacterial strain resuscitation

The biofilm-producing *K. pneumoniae* strains preserved in -70°C glycerol broth were inoculated onto blood agar plates and incubated at 37°C for 18 hours for resuscitation. A single colony was picked and re-inoculated onto a blood agar plate, incubated at 37°C for 18 hours for further use.

### Semi-quantitative crystal violet staining method to determine the amount of biofilm formation

Forty-four overnight culture strains mentioned above (K1-K44) were suspended in sterile saline to a concentration of 0.5 McFarland turbidity standard, and then diluted with double LB(Luria-Bertani) broth to a concentration of 10^6^CFU/ml, which was added to a 48-well flat-bottomed plastic tissue culture plate, with treatment groups including TRQ group, IPM group, TRQ+IPM group, and a control group without medication, as well as a blank group as negative control. After incubation at 37°C for 48 hours, the wells were washed three times with phosphate-buffered saline (PBS, pH 7.4) to remove planktonic bacteria. Each well was then fixed with 2.5% glutaraldehyde for 15 minutes, followed by washing and drying with distilled water. Subsequently, each well was stained with a 1% crystal violet solution for 10 minutes at room temperature, washed three times with double-distilled water to remove excess stain, and finally eluted with ethanol absolute. The absorbance value (A) at OD570nm was measured using a microplate reader. The optical density cut-off (ODc) was declared as three standard deviations above the mean OD of the negative control. Bioflm formation was recorded as follows: non-bioflm forming (A_570_≤1); weak (1<A_570_≤2); moderate (2<A_570_≤3); strong (A_570_>3).

### Scanning electron microscopy to observe changes in biofilm structure

The strong biofilm-producing strain K10 isolated from BALF was selected to observe changes in biofilm structure. Following the same method as semi-quantitative crystal violet staining, a sterilized coverslip was placed at the bottom of the 48-well plate as a biofilm growth carrier and incubated at 37°C for 72 hours to obtain more stable biofilm structure for electron microscopy observation. The carrier was removed, washed with PBS to remove surface bacteria, fixed with 2 ml of 2.5% glutaraldehyde solution for 4 hours, post-fixed with 1% osmium tetroxide for 1 hour, dehydrated with alcohol, sputter-coated in an ion sputter, and finally imaged using a JSM-IT700HR scanning electron microscope produced by JEOL to observe the structure of the biofilm.

Plate counting method to determine the number of live bacteria in the biofilm (Herigstad et al., 2001).

All the 44 strains were cultured to detect the live bacteria in biofilms. A sterile coverslip was placed in a 48-well plate as a carrier, and after incubation for 72 hours, the carrier was removed and washed with sterile PBS to remove planktonic bacteria. The coverslip was then placed in a sterile test tube containing 1 ml of sterile saline, vortexed to release and disperse the bacteria within the biofilm, and the bacterial suspension was diluted to different concentrations with sterile saline. Ten microliters of bacterial suspension at each concentration were evenly spread onto nutrient agar plates and incubated at 37°C for 24 hours for colony counting. Colony counts were expressed as log10(cfu/mL).

### Vibrio harveyi bioluminescence detection method

To determine the effect of TRQ the activity of AI-2, 10 K*. pneumoniae* (K1,4,5,9,10,16,26,32,39, and44) isolated from different specimens were selected. *V. harveyi* BB170 was used as a reporter strain to detect the activity of extracellular AI-2 molecules in *K. pneumoniae*. *K. pneumoniae* cultured at 37°C for 18 hours was suspended in sterile saline to a concentration of 0.5 McFarland turbidity standard and diluted to 10^6^ CFU/ml. One milliliter of bacterial suspension was taken in a 1.5 ml centrifuge tube, centrifuged at 12000 rpm for 10 minutes, and the supernatant was collected and stored. *V. harveyi* BB170 was inoculated into AB(Autoinducer bioassay) medium and cultured at 30°C for 18 hours, suspended in sterile saline to a concentration of 0.5 McFarland turbidity standard, and diluted to a concentration of 10^6^ CFU/ml. The *V. harveyi* suspension was then further diluted at a ratio of 1:5000. Ten microliters of collected supernatant were added to 90 microliters of *V. harveyi* BB170 bacterial suspension, incubated at 30°C for 5 hours, and the luminescence intensity was measured using a multi-function fluorescence microplate analyzer. AI-2 signal molecule activity was expressed as luminescence value as described previously (Zuo et al., 2023). The ratio of luminescence value between the experimental group and the blank control group was calculated as relative luminescence value.

### RT-qPCR determination of biofilm formation-related genes

To detect the effect of TRQ on the biofilm formation-related genes of *K. pneumoniae*, 5 strains(K1,4,5,9,10) were selected. Biofilm formation-related genes included *luxS*, *wbbm*, *wzm*, and *lsrK*. RNA was extracted from *K. pneumoniae*, reverse transcribed into cDNA, and RT-PCR was performed using the reverse transcribed cDNA as template. The melting curve was analyzed to confirm the specificity of amplification, and finally, the relative expression levels of each gene’s mRNA compared to 16S rRNA were calculated using the 2^–ΔΔct^ method as described previously (De Araujo et al., 2010; Fang et al., 2021). The primer sequences were shown in **Table 2**.

**Table 2:** The sequence of primers used in RT-PCR.

Gene name	Primer sequence (5’-3’)	Target gene
RT-luxS-5’	GGAACGCGGTATCCACAC	*luxS*
RT-luxS-3’	TGAGCTCCGGGATCTGGT	
RT-lsrK-5’	CAGGGTACCGGTCTCTTTGA	*lsrK*
RT-lsrK-3’	TACGAGGTCTCGGGACAAAC	
RT-wbbM-5’	TTATCAGGCTGCCATTGCCAT	*wbbM*
RT-wbbM-3’	CAGCTATATGCCCAATAACGC	
RT-wzm-5’	CTATCGAAGACGTATCCTTTAC	*wzm*
RT-wzm-3’	ATATTCTCACGCCCGGTAAG	
16S-rRNA-5’	ATGACCAGCCACACTGGAAC	16S
16S-rRNA-3’	CTTCCTCCCCGCTGAAAGTA	

### Construction of a guinea pig lung infection model with *K. pneumoniae* biofilm

To evaluate the antibacterial effect of TRQ against *K. pneumoniae in vivo*, the strongest biofilm formation strain (K21) isolated from BALF was selected to construct the guinea pig lung infection model. Female guinea pigs of the British-Dutch hybrid strain, weighing between 200-300g, were randomly divided into drug treated groups concluding TRQ group, IPM group, and TRQ+IPM group, and sterile saline group as the control, with 8 guinea pigs in each group. All animals were pretreated with subcutaneous injection of dexamethasone at a dose of 3 mg/(kg·d) once daily for 7 consecutive days to suppress their immune function. On the 8th day, each animal received intranasal administration of 200 microliters of bacterial suspension containing 5×10^8^ CFU/ml of *K. pneumoniae(*K21) for three consecutive days. Treatment groups received intravenous administration of drugs every 12 hours through the lateral tail vein, with TRQ group receiving TRQ at a dose of 0.5 ml/kg, IPM group receiving IPM at a dose of 15 mg/kg, and control group receiving an equal volume of sterile saline. Medication was continued for 14 consecutive days. On days 7 and 14 after treatment, 8 animals from each group were euthanized, and lung tissues were surgically removed for observation of pathological changes through HE staining and determination of viable bacterial counts within the lung tissues.

### Determination of viable bacterial counts in lung tissues

A small piece of lung tissue was aseptically excised, weighed, placed in a tissue homogenizer with 1 ml of sterile saline to make a tissue suspension, which was then serially diluted with sterile saline. Ten microliters of each dilution were inoculated onto nutrient agar plates and incubated overnight at 37°C. Colony counts were calculated using the lowest countable dilution factor, with bacterial load in lung tissue (cfu/g) calculated as (colony count×dilution factor/lung tissue weight).

### Pathological examination

On the day of tissue collection, lung tissue samples were surgically removed, fixed with 2.5% glutaraldehyde solution, embedded in paraffin, sectioned at 6 micrometers thickness, stained with HE, and observed under a microscope.

### Statistical methods

Data obtained from this experiment are expressed as Mean ± SEM and analyzed using GraphPad Prism software through t-tests and one-way analysis of variance (ANOVA) to assess differences between groups. A p-value less than 0.05 was considered statistically significant. ***indicates p<0.0001; **indicates p<0.001; *indicates p<0.05.

## Results

### The amount of biofilm formation

**Figure 1:**
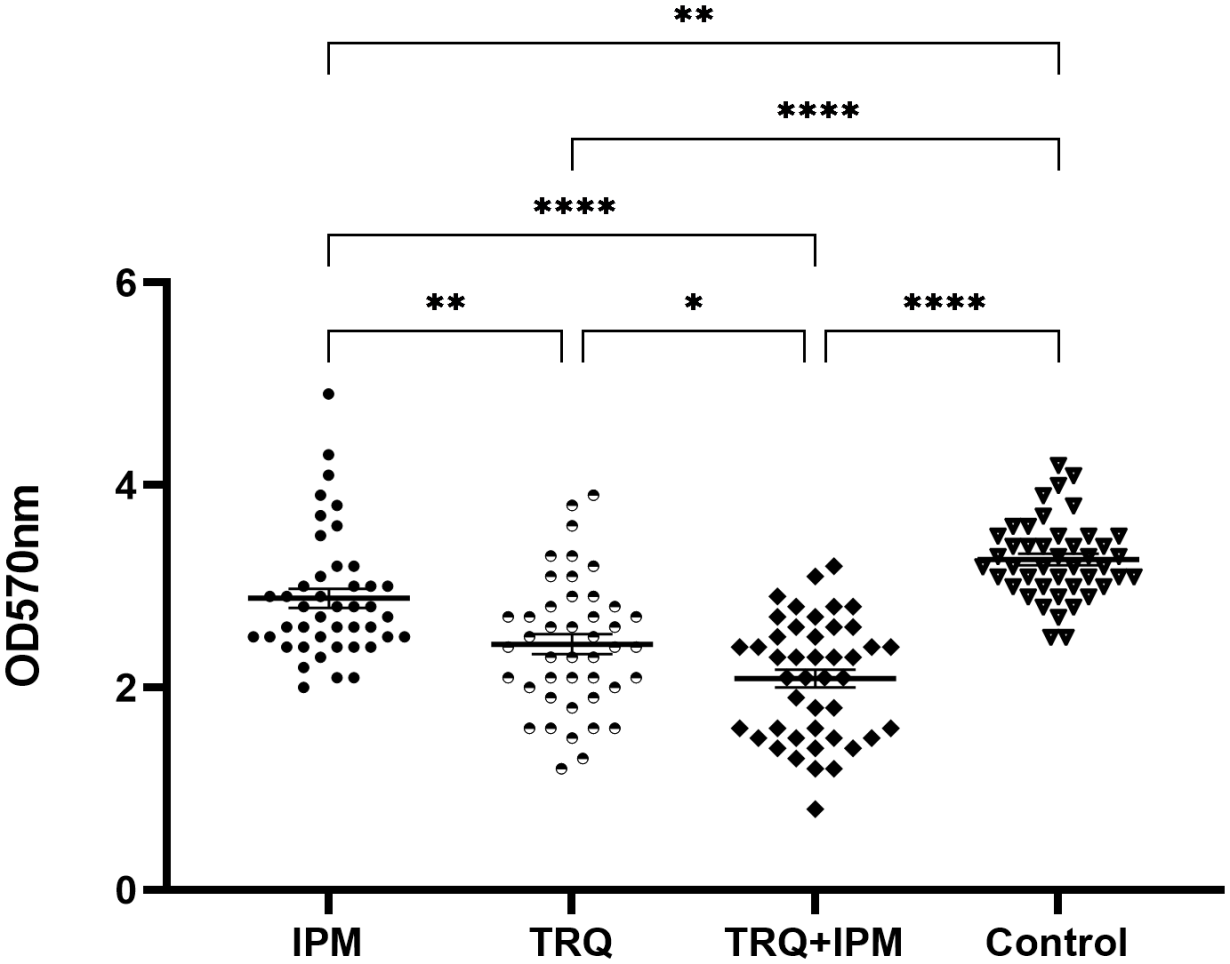
Effects of TRQ group, IPM group, and combined TRQ+IMP group on the amount of *K. pneumoniae* biofilm formation. ***indicates p<0.0001; **indicates p<0.001; *indicates p<0.05.

The drug concentration in the TRQ group was 1/2MIC of TRQ, the IPM group was 1/2MIC of IPM, and the TRQ+IPM group was 1/2MIC of IPM and 1/2MIC of TRQ. The results are shown in **Figure 1**. Compared with the control group, the amount of *K. pneumoniae* biofilm in the TRQ group, IPM group, and TRQ+IPM group was significantly reduced (p<0.05); meanwhile, compared with the IPM group, the amount of *K. pneumoniae* biofilm produced in the TRQ group and the TRQ+IPM group was significantly reduced (p<0.05).

### Changes in the structure of *K. pneumoniae* biofilm

**Figure 2:**
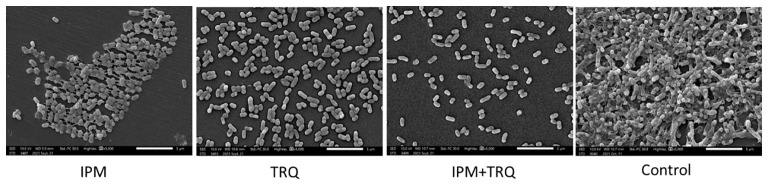
The effect of TRQ group, IPM group, and TRQ+IPM group on the structure of *K. pneumoniae* biofilm.

The effect of TRQ on the morphology and structure of *K. pneumoniae* biofilm was observed by scanning electron microscopy. The results are shown in **Figure 2**. Compared with the control group, the bacterial density within the *K. pneumoniae* biofilm in the TRQ group and the IPM+TRQ group was significantly reduced, and the biofilm structure was loose and the thickness was reduced. Among them, the number of bacteria in the biofilm of the IPM+TRQ group was not only greatly reduced, but also the colonies were scattered; compared with the control group, although the thickness of the biofilm in the IPM group was also reduced, and the amount of bacteria was also reduced, there were obvious membranous substances between the colonies, which adhered the colonies into clumps.

### The number of viable bacteria in the biofilm

**Figure 3:**
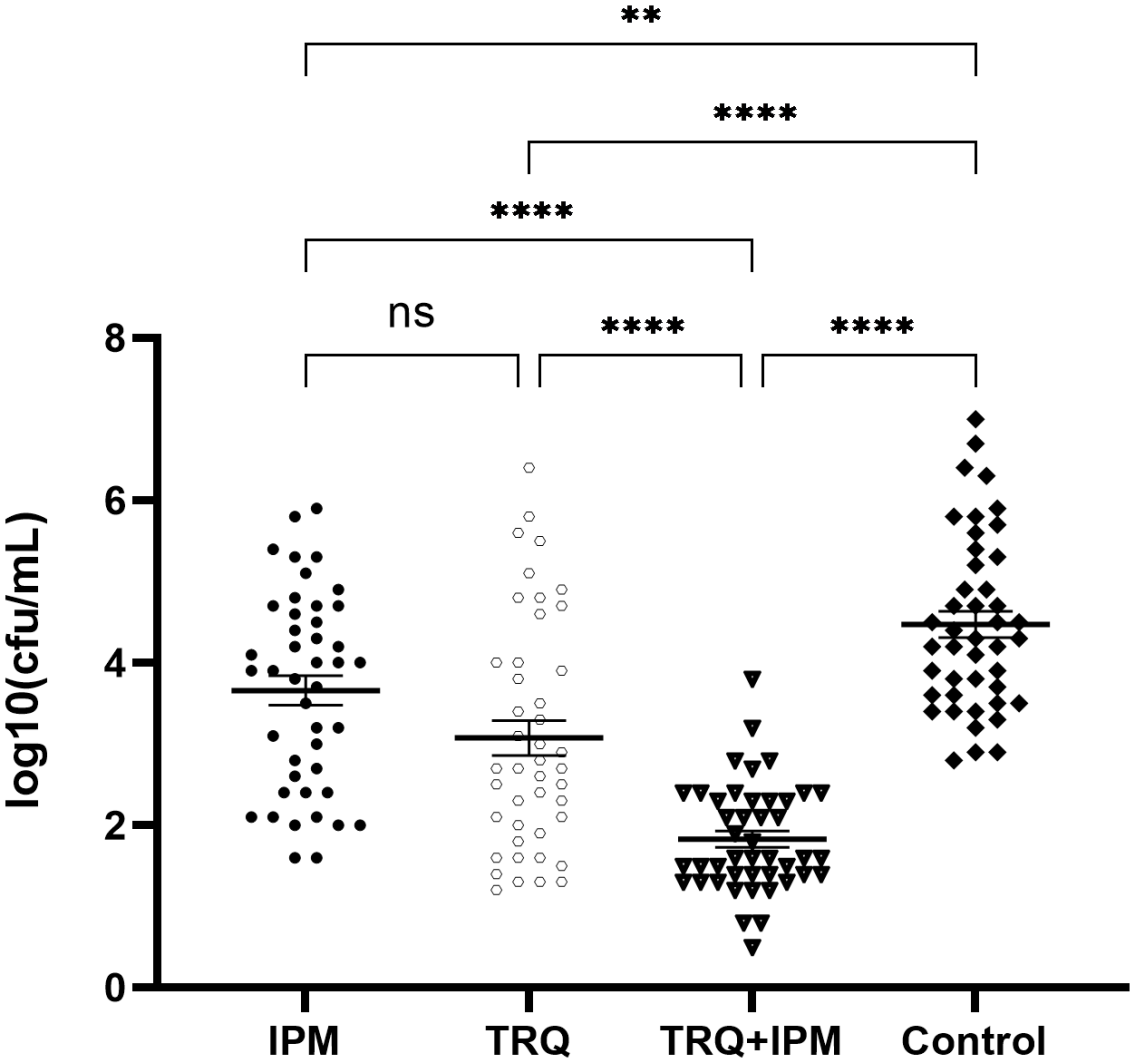
The effect of TRQ on the number of viable bacteria in the biofilm. ***indicates p<0.0001; **indicates p<0.001; *indicates p<0.05.

Compared with the control group, the number of viable bacteria in the biofilm of the TRQ+IPM group, TRQ group, and IPM group significantly decreased, with statistical significance; among them, the number of viable bacteria in the biofilm of the TRQ+IPM group was significantly lower than that of the TRQ group. Compared with the TRQ group, there was no significant difference in the number of viable bacteria in the biofilm of the IPM group, with a p-value of 0.074. The results are shown in **Figure 3**.

### Detection of AI-2 activity

**Figure 4:**
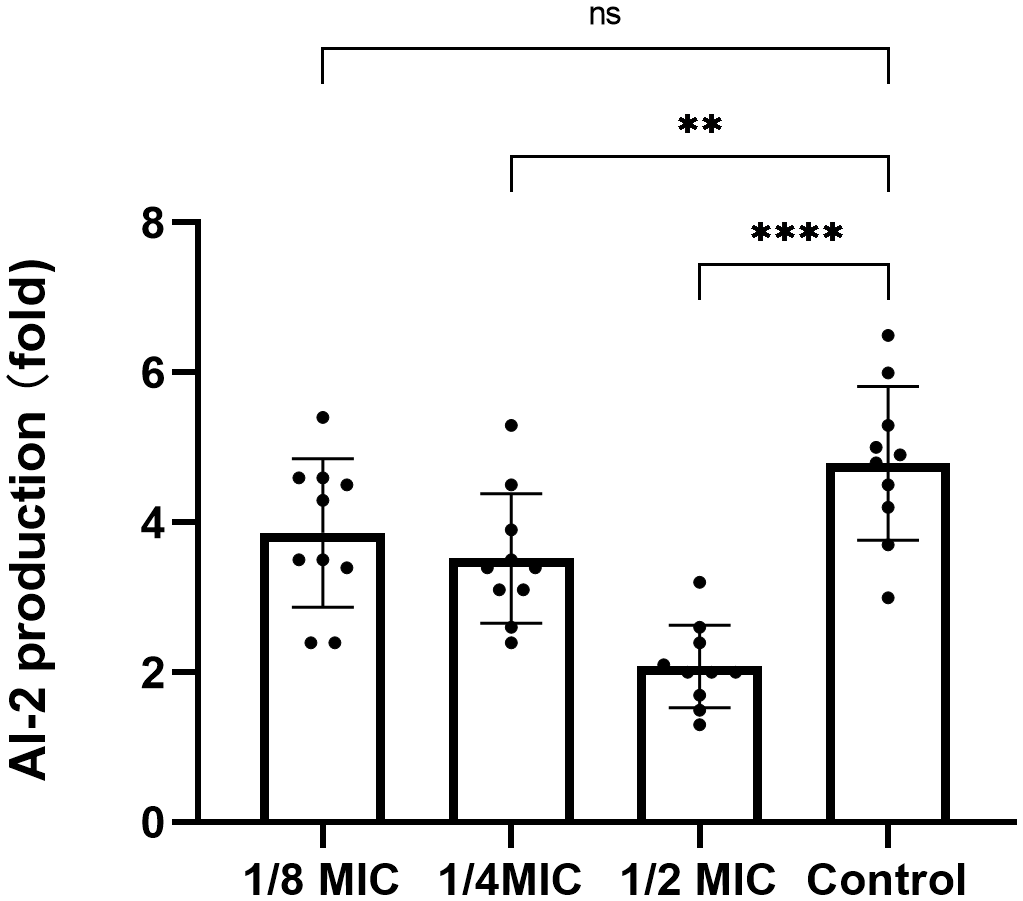
The effect of different concentrations of TRQ on AI-2 activity in *K. pneumoniae*. ***indicates p<0.0001; **indicates p<0.001; *indicates p<0.05.

Compared with the control group, the relative fluorescence values of AI-2 detected in the TRQ groups at 1/2MIC and 1/4MIC were significantly lower than those in the control group, which was statistically significant. There was no significant difference in the relative fluorescence values detected between the TRQ group at 1/8MIC and the control group (**Figure 4**).

### Expression of biofilm-related genes

**Figure 5:**
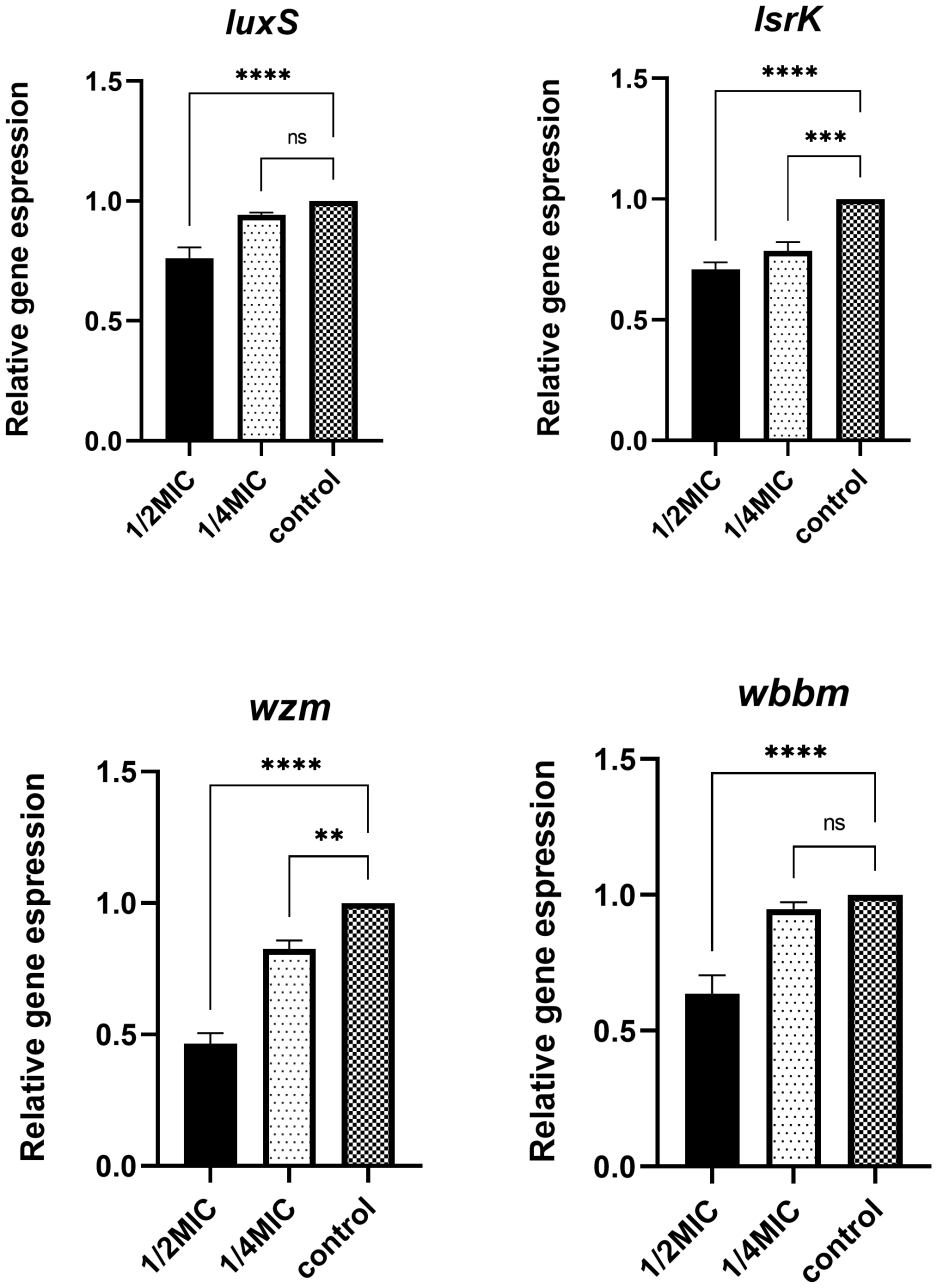
Differential expression of biofilm-related genes after treatment with different concentrations of TRQ. ***indicates p<0.0001; **indicates p<0.001; *indicates p<0.05.

*K. pneumoniae* was treated with TRQ at concentrations of 1/2MIC and 1/4MIC, respectively. As shown in **Figure 5**, compared with the control group, TRQ at 1/2MIC significantly reduced the expression of *luxS*, *wbbm*, *wzm*, and *lsrK* genes in *K. pneumoniae*, with statistical significance. TRQ at 1/4MIC significantly reduced the expression of *wzm* and *lsrK* genes, with statistical significance, and also reduced the expression of *luxS* and *wbbm* genes to some extent, but there was no statistical significance, with p-values of 0.244 and 0.597, respectively.

### Results of guinea pig lung tissue HE staining

**Figure 6:**
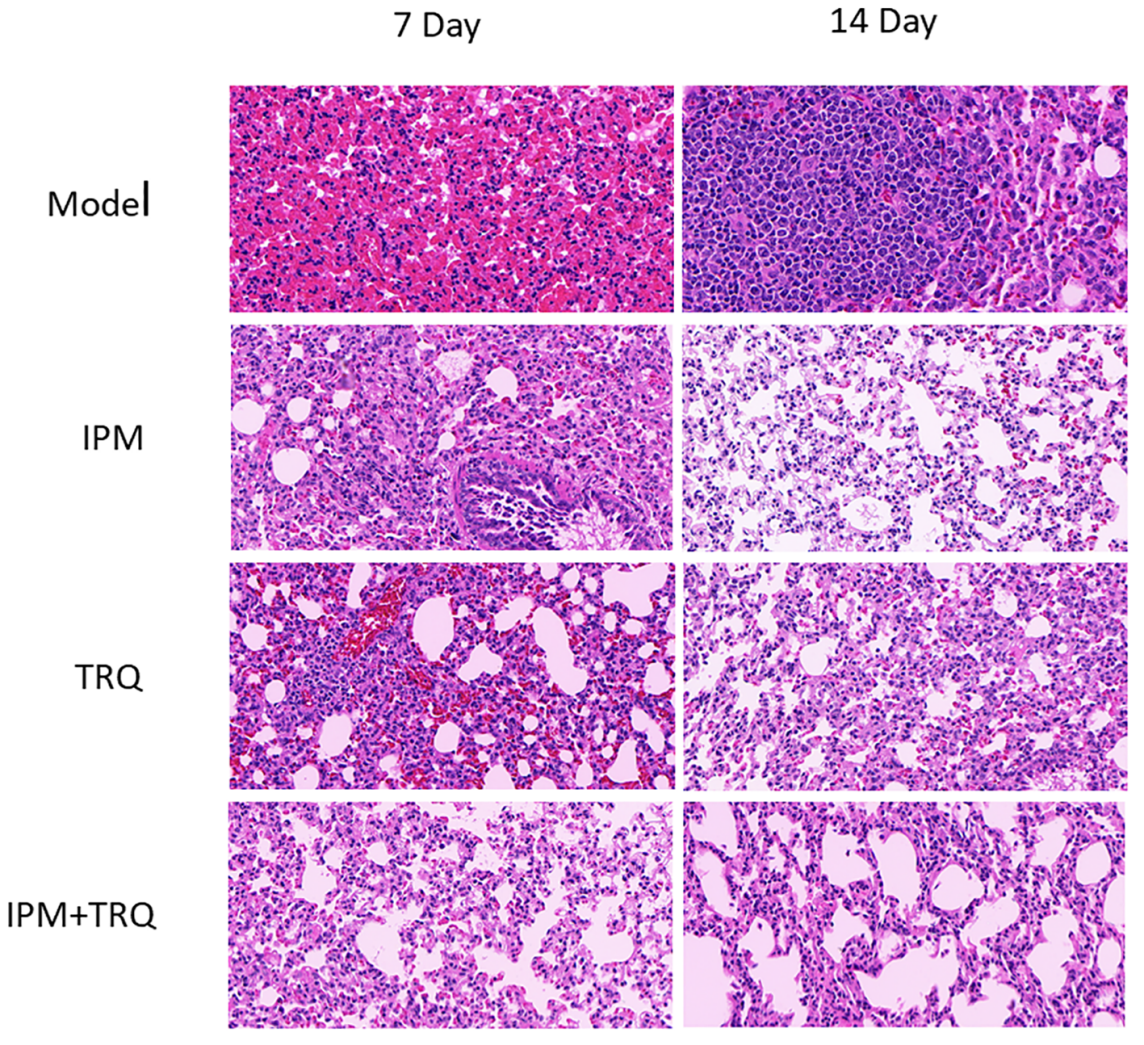
Results of guinea pig lung tissue HE staining.

In the model group, after 7 days of infection, the lung tissue showed alveolar collapse, increased alveolar wall thickness, severe inflammatory cell infiltration, and alveolar wall vascular congestion. After 14 days, inflammatory cells aggregated to form granulomatous nodules, with alveolar fusion and unclear structure. In the IPM and TRQ groups, after 7 days of treatment, there was obvious infiltration of inflammatory cells and red blood cells around the blood vessels, with vascular congestion. After 14 days of treatment, the inflammatory cell infiltration in the lung tissue decreased. In the IPM+TRQ group, after 7 days of treatment, there was a significant reduction in inflammatory cells in the lung tissue, with mild focal inflammation observed. After 14 days, a small amount of inflammatory cells were seen in the alveolar cavity, with clear alveolar boundaries. The results are shown in **Figure 6**.

### Determination of viable bacteria count in lung tissue

**Figure 7:**
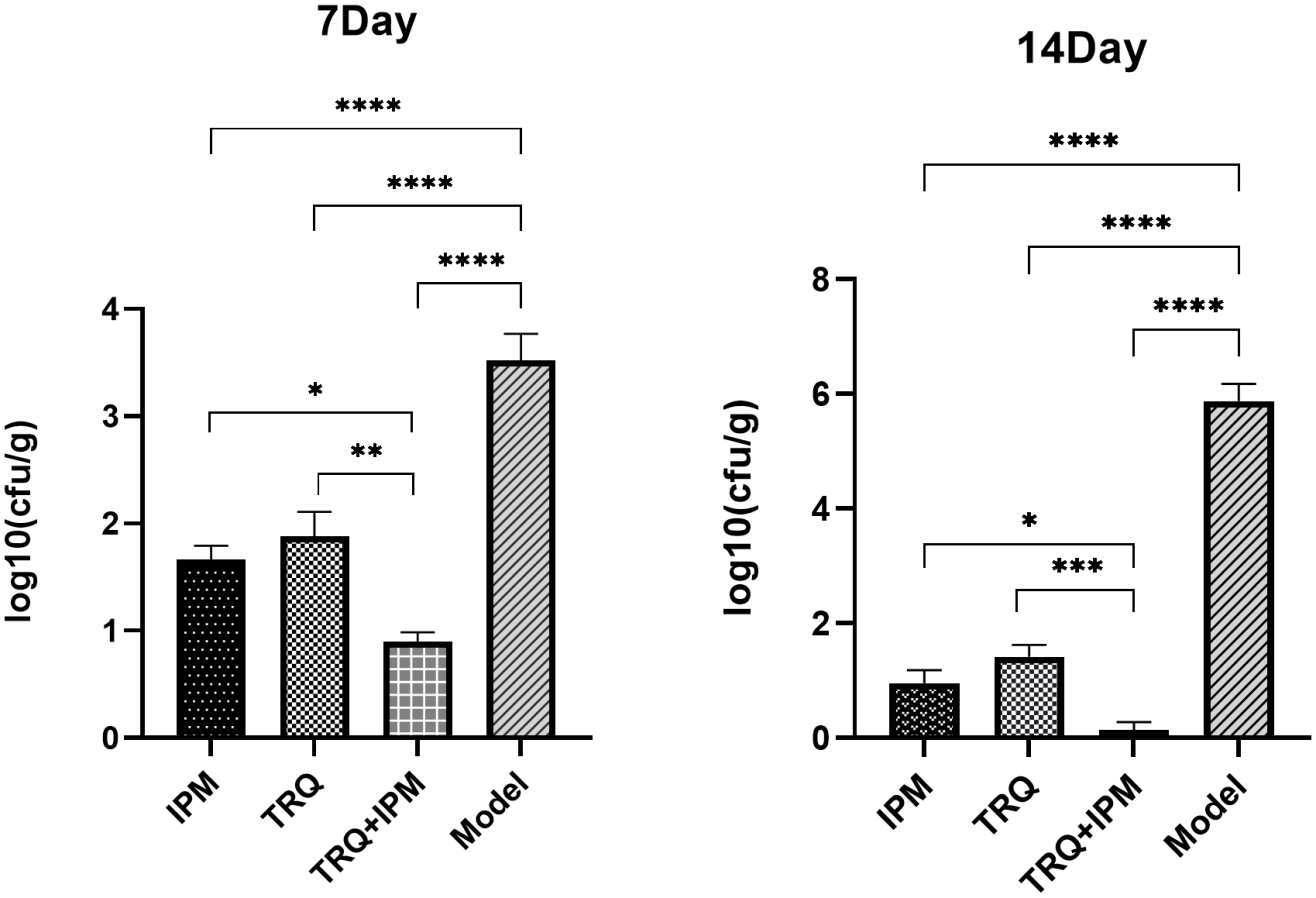
Bacterial load in lung tissue on the 7th and 14th days after treatment. ***indicates p<0.0001; **indicates p<0.001; *indicates p<0.05.

After 7 days and 14 days of treatment, the number of bacteria in the lung tissue of guinea pigs in the control group was significantly higher than that in the drug treatment groups, respectively. And the number of viable bacteria in the lung tissue of the IPM+TRQ group was significantly lower than that in the IPM and TRQ monotherapy groups. The results are shown in **Figure 7**.

## Discussion

In recent years, infections caused by *K. pneumoniae*, especially those producing carbapenemases, have caused widespread epidemics around the world (Nordmann et al., 2009; Castanheira et al., 2020; Wang et al., 2020a). Due to the limited number of clinically available antimicrobial agents, infections caused by such bacteria are often associated with high mortality rates, posing significant difficulties and challenges to clinical anti-infective treatment. Therefore, it is urgent to find effective and non-resistant antimicrobial agents to meet the growing demand for the treatment of infections caused by resistant bacteria.

Numerous trial studies have shown that TRQ has certain advantages in the treatment of severe pneumonia caused by resistant bacteria. The combination of conventional antibiotics and TRQ can significantly improve pathogen clearance, reduce the detection of drug-resistant bacteria during treatment (Zhou et al., 2021), diminish inflammatory reactions, and exhibit good safety (Wang et al., 2020b). Studies have also shown that pneumonia and respiratory tract infections are closely related to the formation of biofilms on the surface of the respiratory tract (Assefa and Amare, 2022; Dargahi et al., 2022).

Bacterial biofilm is a specific phenomenon that occurs during the growth of bacteria in groups. Biofilms not only enhance the ability of bacteria to colonize the body, but also cause resistance by preventing antimicrobial agents from binding to bacterial target sites (Li and Ni, 2023). At the same time, the efficient transfer of resistant plasmids within bacterial biofilms is also the main way for bacteria to acquire resistance genes, and with the dissociation of biofilms, resistant genes are continuously disseminated (Bowler et al., 2020). In addition to inducing bacterial resistance, biofilms can also evade the attacks from the body’s immune system through the shielding effect of their own extracellular matrix and the secretion of regulatory factors, further evolving into drug-resistant bacteria. Therefore, bacteria present in biofilms can resist the killing of antibiotics and promote the formation of drug resistance, which is an important reason for chronic persistent infections (Surgers et al., 2019). *K. pneumoniae* has a strong ability to produce biofilms, and resistant strains can form bacterial biofilms more easily (Dan et al., 2023). Therefore, the development of resistance in *K. pneumoniae* is related to the formation of biofilms (Shein et al., 2022).

We hypothesize that TRQ’s therapeutic effect on biofilm-related infections is related to its inhibitory effect on biofilms. To confirm the above viewpoint, we used semi-quantitative crystal violet staining to determine the amount of biofilm formation, scanning electron microscopy to observe the structure of bacterial biofilms, and plate counting to detect the number of bacteria in the biofilm. The results showed that TRQ had a significant inhibitory effect on the production of *K. pneumoniae* biofilm. Although IPM could also inhibit the production of bacterial biofilm, TRQ had a greater inhibitory effect on bacterial biofilm than IPM, and the effect was more pronounced. Under the scanning electron microscope, it was observed that although the amount of bacteria in the IPM treatment group was reduced, there were membrane-like substances connecting the bacteria, while in the TRQ group, no obvious membrane-like substances were seen between the colonies, and the colonies appeared scattered (**Figure 2**). It is speculated that the inhibitory effect of IPM on biofilm may be due to its bactericidal effect on *K. pneumoniae*, which leads to a reduction in the amount of bacteria, thereby reducing biofilm production. In the test results of the number of bacteria in the biofilm, we found that although both TRQ and IPM can reduce the number of bacteria in the biofilm, if they are used in combination, they can greatly enhance the clearance rate of *K. pneumoniae* producing strong biofilms (**Figure 3**). Guinea pig lung infection experiments also showed that TRQ can effectively reduce the aggregation of inflammatory cells and the extravasation of red blood cells, and reduce the inflammatory response of lung tissue. Meanwhile, the combination of TRQ and IPM can enhance the anti-infective effect of TRQ and greatly reduce the number of viable bacteria in lung tissue (**Figures 6**, **7**). This further confirms that TRQ can enhance the killing effect of antibiotics on *K. pneumoniae* by inhibiting the formation of bacterial biofilms, thereby exerting an anti-infective effect.

However, the mechanism of how TRQ inhibits the formation of bacterial biofilm is still unclear. Studies have shown that the AI-2 quorum sensing system mediated by the *LuxS* gene can promote the formation and maturation of biofilm by facilitating intercellular communication (Sun et al., 2020). The *luxS*/AI-2 QS system was previously identified in *K. pneumoniae*, where *luxS* was shown to be critical for AI-2 synthesis, and the expression level of *LuxS* is related to the ability in biofilm formation of *K. pneumoniae* (Chen et al., 2020). Meanwhile, the expression of *luxS* is constitutive and the peaks of AI-2 production and transcriptional level of *luxS* appear at the same time point, which prove that the transcription of *luxS* is tightly correlated to AI-2 production in *K. pneumoniae* (Zhu et al., 2011).These facts support the idea that the quorum sensing is *luxS* dependent in *K. pneumoniae*. According to the result of our study, TRQ could significantly inhibit the expression of *LuxS* in *K. pneumoniae* at 1/2MIC concentration, but not at 1/4MIC concentration. This proved that the inhibitory effect of TRQ on the expression of *LuxS* is concentration dependent. Meanwhile, *V. harveyi* bioluminescence assay showed that TRQ at 1/2MIC and 1/4MIC could significantly inhibit the AI-2 signaling. That indicated that TRQ at 1/4MIC could significantly inhibit the AI-2 signaling but not down-regulate the expression of *LuxS*, which give us the message that maybe other inhibition mechanism of TRQ on the AI-2 signaling exist in addition to down-regulate the expression of *LuxS.* Anyhow, we speculate that the inhibitory effect of TRQ on *K. pneumoniae* biofilm may be related to quorum sensing system.

Additionally, study have identified that *K. pneumoniae* promotes bacterial biofilm formation through the lipopolysaccharide synthesis and mutations in QS related genes induced changes in biofilm formation and LPS synthesis. And gene *wbbM* and *wzm* are two LPS-synthesis-related genes involved in the synthesis of bacterial lipopolysaccharide, which are related to the formation of bacterial biofilm (De Araujo et al., 2010; Ernst et al., 2020). In our study, the result of q-RT PCR also showed that TRQ could significantly down-regulate the expression of gene *wbbM* and *wzm* in *K. pneumoniae*, which consistent with the observation that TRQ could inhibit the biofilm formation of *K. pneumoniae.*

Meanwhile, our study also showed that TRQ could down-regulate the gene *lsrK*. The *lsrK* gene is located upstream of the *lsr* operon and its transcription is regulated by the *lsrRK* operon. As a phosphorylated kinase of the AI-2 signaling molecule, *LsrK* is involved in the phosphorylation process of the AI-2 signaling molecule. The phosphorylated AI-2 molecule binds to the repressor *LsrR*, which causes it to be released from the *lsr* operon, activating the transcription of *lsr* and *lsrRK*. Therefore, the *lsrK* gene plays an important role in regulating the activity and stability of the intracellular AI-2 signaling pathway, and inhibiting *LsrK* can reduce the pathogenicity of pathogenic bacteria by interfering with the quorum sensing system signal (Medarametla et al., 2021; Shi et al., 2023). In this study, we found that TRQ at concentrations of 1/2MIC and 1/4MIC could significantly inhibit the activity of AI-2 molecules in *K. pneumoniae* (**Figure 4**). Therefore, it is possible that TRQ inhibits the activity of AI-2 molecules, thereby reducing phosphorylated AI-2, leading to inhibition of the activation of *lsr* and *lsrRK* transcription, thereby inhibiting the expression of *lsrK*, which is consistent with the results of this experiment. It can be seen that *luxS*, as a higher-order regulatory gene, together with genes such as *wbbM*, *wzm*, *lsrK*, and AI-2 molecular signals, affects the formation of biofilm.

## Conclusion

Our study found that the combination of TRQ and IPM had a synergistic bactericidal effect on *K. pneumoniae* in an *in vivo* experiment of a guinea pig lung infection model. Additionally, we have confirmed that TRQ can effectively inhibit the formation of *K. pneumoniae* biofilm *in vitro*, and `down-regulated the expression of bacterial biofilm-related genes and the activity of the quorum-sensing molecule AI-2. Therefore, we speculate that TRQ inhibits the formation of bacterial biofilm by blocking the quorum sensing system, thereby enhancing the bactericidal effect of antibiotics on bacteria and reducing drug resistance. These new evidences might contribute to the selection of TRQ to the therapeutic treatments of infections caused by *K. pneumoniae*, particularly in combination with conventional antibiotics.

## Data availability statement

The raw data supporting the conclusions of this article will be made available by the authors, without undue reservation.

## Ethics statement

The animal study was approved by Ethics Committee of Zhoupu Hospital in Pudong New Area, Shanghai. The study was conducted in accordance with the local legislation and institutional requirements.

## Author contributions

WZ: Writing – original draft, Writing – review & editing. MH: Formal Analysis, Writing – original draft. NK: Writing – review & editing. YN: Writing – original draft. AL: Writing – original draft. YY: Writing – original draft.
